# Protocol for validating drug efficacy and safety in scalable 96-well platforms of human cortical organoids and melanoma brain metastases

**DOI:** 10.1016/j.xpro.2026.104568

**Published:** 2026-05-19

**Authors:** Kim Krieg, Fatima-Zahra Rachad, Annika Wittich, Pia Kauven, Arjen Weller, Ole Pless

**Affiliations:** 1 Fraunhofer Institute for Translational Medicine and Pharmacology ITMP, Discovery Research ScreeningPort, 22525 Hamburg, Germany

**Keywords:** Cell culture, Cell-based Assays, Cancer, High Throughput Screening, Cell Differentiation, Organoids

## Abstract

Metastatic colonization at secondary organs, such as the brain, represents a critical therapeutic window in cancer progression. Here, we present a protocol for a scalable *in vitro* screening platform for melanoma brain metastases. We describe steps for generating human cortical organoids in 96-well plates, followed by co-culture with melanoma cells to mimic the colonization process. This platform enables quantitative assessment of drug-induced metastasis regression and safety testing using fluorescence and luminescence readouts, targeting colonized melanoma cells in the brain microenvironment.

For complete details on the use and execution of this protocol, please refer to Krieg et al.^1^

## Highlights

• Instructions for modeling the colonization process of human brain metastasis *in vitro*
• Steps for co-cultivation of human cortical organoids and melanoma cells
• Procedures for reproducible fluorescence- and luminescence-based screening assays
• Guidance on anti-metastatic activity and drug safety testing

## Abstract (editor highlights)

Publisher’s note: Undertaking any experimental protocol requires adherence to local institutional guidelines for laboratory safety and ethics.

## Graphical abstract

**Figure:**
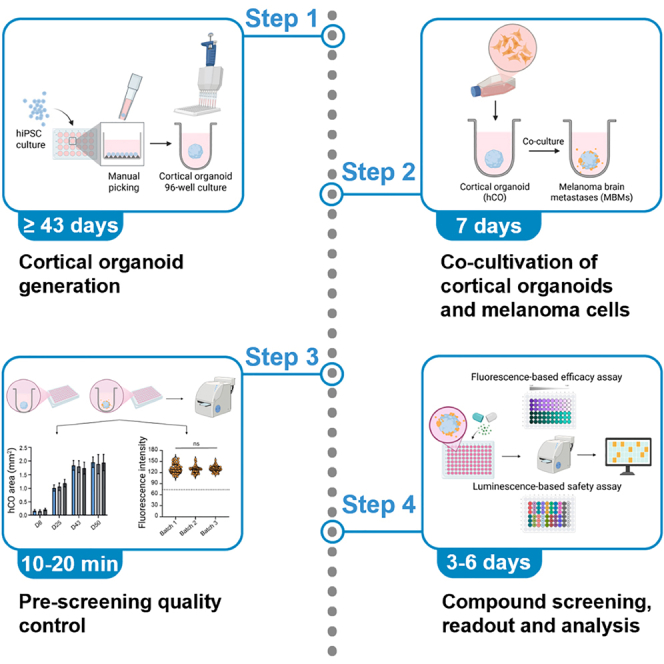

## Before you begin

Metastatic organ colonization, the progression from disseminated cancer cells (DCCs) to micrometastases at distant organs, is considered the rate-limiting step of the metastatic cascade and offers a pivotal therapeutic window.^2^^,^^3^^,^^4^^,^^5^ Brain metastasis occurs in at least 20% of cancer patients and presents a significant clinical challenge.^6^^,^^7^ Although DCCs undergo significant molecular evolution and phenotypic adaptation during metastatic progression, brain metastases are still mainly treated using therapies designed against primary tumors.^8^^,^^9^^,^^10^^,^^11^^,^^12^ The limited efficacy of currently available therapies in reliably preventing disease progression and recurrence,^5^^,^^13^^,^^14^^,^^15^ suggests that the central nervous system (CNS) microenvironment is an essential component in drug discovery against brain metastases. To overcome the unmet clinical need for tailored experimental therapies, this protocol aims for the development of a human preclinical model to support metastasis-integrating drug discovery. The procedure is based on human induced pluripotent stem cell (hiPSC)-derived *in vitro* models, which are powerful tools for recapitulating key features of human organs, particularly cortical tissue^16^^,^^17^ affected by brain metastasis.^18^^,^^19^^,^^20^ We have developed a screening-compatible platform of human melanoma brain metastases (MBM), which provides a valuable tool to target melanoma plasticity and early colonization events within the CNS microenvironment.^1^ This platform enables validation and prioritization of drug candidates with respect to metastatic regression and CNS drug safety.

### Innovation

Unlike conventional *in vitro* models of the primary tumor, this multicellular three-dimensional MBM screening platform mimics key features of metastatic colonization, offering the opportunity to specifically target colonizing (patient-derived) melanoma cells in a human brain niche context. By implementing standardized parameters in a 96-well format, this protocol achieves high scalability and consistency in model generation and assay performance, meeting stringent demands for drug testing. The miniature screening assay enables quantitative assessment of drug-induced metastatic regression and neurotoxicity assessment in both MBM models and human cortical organoids (hCOs), with high reproducibility and fast data acquisition. Together, the platform provides a promising preclinical drug discovery tool for identifying and prioritizing (repurposing) drug candidates for future clinical testing.

### Institutional permissions

The ethical consent for all hiPSC lines in this study was granted by the respective institutions as described in the initial publications.^21^^,^^22^^,^^23^ Permission to utilize patient-derived specimens for this protocol is required by the medical ethics committee of the University of Regensburg, i.e., for patient-derived disseminated cancer cells.

### Maintenance culture of human induced pluripotent stem cells

**Timing: Variable**

This section provides information about the preparation of human hiPSCs for subsequent differentiation. We utilized high-quality cryostocks containing hiPSC colonies of well-characterized human wild-type hiPSC cell lines that were routinely tested for absence of mycoplasma contamination. All cell culture work needs to be conducted under aseptic conditions within a laminar flow hood.

***Note:*** The protocol was established with the hiPSC cell line WISCi004-B and validated using ZIPi013-B and HHUUKDi009-A.

1. Thaw and culture hiPSC colonies.
  ***Note:*** For cryopreservation, hiPSC colonies from one 6-well were distributed equally into two cryovials. Consequently, each vial is thawed and seeded at half the standard maintenance splitting ratios.
  a. Coat 6-well plates with Matrigel.
    ***Note:*** We recommend thawing Matrigel on ice, dilute in ice-cold DMEM/F12 medium to a concentration of 40 ng/mL and incubate 2 mL/well at 37°C for 1 h.
  b. Prepare mTeSR Plus medium by mixing the basal medium and supplement provided by the kit following the manufacturer’s instructions and warm at room temperature (RT, 20°C–25°C).
  c. Remove Matrigel from the 6-well plates and add 2 mL mTeSR Plus medium to each well prior to thawing.
    **CRITICAL:** Dissolve Y-27632 following the manufacturer’s instruction and supplement medium with 10 μM for the initial 24 h of cell culture after thawing.
  d. Thaw a cryovial of hiPSC colonies.
    i. Transfer the cryovial to a 37°C water bath for 1 to 2 min.
    ii. Transfer the cell suspension to a falcon tube and add 9 mL prewarmed DMEM/F12 medium.
    iii. Centrifuge at 300 × *g* for 5 min and remove the supernatant.
    iv. Resuspend cell pellet carefully in 1 mL of mTeSR Plus medium supplemented with 10 μM Y-27632 and seed corresponding volumes of cell suspension into prepared 6-well plates.
      **CRITICAL:** Gentle resuspension protects the intactness of colony structure.
      ***Note:*** We recommend seeding the content of one cryovial in ratios 1:10–1:40.
    v. Allow cells to settle for 24 h in the incubator at 37°C.
  e. Change media the next day and then every other day using mTeSR Plus medium.
  f. Passage hiPSC cultures at approximately 50–70% confluency every four to five days.
    i. Wash each well with D-PBS and incubate in 1 mL Gentle Cell Dissociation Reagent for approximately 4 min at RT (20°C–25°C).
    ii. Aspirate the solution and add 1 mL of mTeSR Plus medium.
    iii. Use a scraper to detach hiPSCs and carefully resuspend to break colonies into smaller pieces.
    iv. Seed colonies at ratios between 1:20 and 1:80 in mTeSR Plus medium in Matrigel-coated 6-well plates.
  g. Regularly observe hiPSC morphology under the microscope.
    **CRITICAL:** To initiate differentiation, hiPSC cultures should reach 60**–**80% confluency. Cells should be passaged at least once after thawing and colonies should be free of any signs of spontaneous differentiation before continuing with the protocol. Depending on the packing density of cell lines, prepare 1 to 2 full 6-well plates of hiPSCs for each hCO batch.
    ***Note:*** For comparative analysis, biological replicates are defined as independent batches of hCOs, derived from separate sources of hiPSCs (i.e., independent thaw or passage).

### Preparation of routine cell cultures of melanoma cells

**Timing: Variable**

In this section, we describe the maintenance of fluorescently labeled cancer cells under standard 2D cell culture conditions prior to co-cultivation. All cell culture work needs to be conducted under aseptic conditions within a laminar flow hood.

**CRITICAL:** The protocol requires intrinsic cellular expression of fluorescence proteins.

***Note:*** In this protocol we describe the approach using melanoma cell line A375 (CRL-1619), stably transduced with a red fluorescent protein (RFP). We also validated the workflow using disseminated cancer cells derived from lymph node biopsies of melanoma patients (referred to as MelDCCs), stably transduced with a green fluorescence protein (GFP). Detailed instructions for isolation and cultivation of MelDCCs can be found in.^1^^,^^24^^,^^25^

2. Thaw and culture A375 melanoma cells.
  a. Prepare A375 Complete Medium and warm at RT (20°C–25°C).
  b. Thaw a cryovial of A375 single cell suspension.
    i. Transfer the cryovial to a 37°C water bath for 1–2 min.
    ii. Transfer the cell suspension to a falcon tube and add 9 mL prewarmed DMEM/F12 medium.
    iii. Centrifuge at 300 × *g* for 5 min and remove the supernatant.
    iv. Resuspend cell pellet carefully in 1 mL of A375 Complete Medium and seed 1 × 10^6^ cells in one T75 flask filled with 12 mL A375 Complete Medium.
    v. Allow cells to settle for 24 h in the incubator at 37°C.
  c. Change medium the next day and then every other day and passage cells every three to four days at a confluency of 70–90%.
    i. Wash each flask with D-PBS and incubate in 5 mL Trypsin-EDTA for approximately 3–5 min at RT (20°C–25°C).
    ii. Detach cells by repetitive rinsing.
    iii. Transfer cell suspension to a falcon tube and add 10 mL DMEM/F12.
    iv. Centrifuge at 300 × *g* for 5 min and remove the supernatant.
    v. Resuspend the supernatant in 1 mL A375 Complete Medium.
    vi. Seed cells in a ratio of 1:10 to 1:20 each T75 flask, filled with 12 mL A375 Complete Medium.
    vii. Allow cells to settle for 24 h in the incubator at 37°C.

**CRITICAL:** Maintain cells in their log growth phase and adapt passaging schedules according to the timeline of organoid culture. One T75 flask with cancer cells need to be ready for passaging at organoid cultivation day 43. Therefore, we recommend thawing A375 cells at the latest at organoid cultivation day 35–37 and passage at least once before usage.

### Preparation of the plate layout

**Timing: ∼ One day**

**CRITICAL:** For statistical robustness, each compound and concentration should be evaluated at least in triplicates and/or biological replicates. A high amount of technical control replicates supports the analysis of assay stability. Do not assign the border wells, which are more likely affected by edge-effects. For exemplary templates see Figure S1.

3. Design the layout for compound testing in 96-well plates.

***Note:*** For example, prepare layouts for single-concentration screening of multiple compounds or multiple-point concentration-response profiling of individual drug candidates.

4. For single-concentration screening, assign controls to the plate layout.

**CRITICAL:** The negative control is the solvent of compounds (e.g., 0.1% DMSO) and the positive control should utilize a physiologically relevant reference compound with known efficacy in the model to define the dynamic range of the assay.

***Note:*** We utilized the combination therapy of encorafenib and binimetinib at 10 μM concentration, as one of the current standards of care.

5. For 10-point concentration-response curves, assign a concentration range covering reported half inhibitory concentrations (IC_50_) or clinically relevant doses.

***Note:*** Start with the highest concentration and include the negative control in the last column.

### Preparation of small-molecule compounds

**Timing: ∼ One day**

**CRITICAL:** Consider proper sourcing, preparation and storage as critical requirements for quality and stability of small molecule compounds. For more information see problem 1. Compound handling may involve the use of hazardous substances, requiring adherence to safety protocols and protective measures.

6. Prepare and store fresh 10 mM compound stocks in DMSO at −20°C for long-term storage.

***Note:*** Use low-binding polypropylene plates and proper plate sealing.

7. Prepare assay-ready plates from source plates or other fresh stocks of compounds in accordance with plate layouts.

**CRITICAL:** Remove plates with the dissolved compound stocks from the freezer and equilibrate to RT (20°C–25°C). Centrifuge compound plates before usage to reduce the risk of spillover and avoid repetitive freeze-thaw cycles.

***Note:*** For concentration-response titration, prepare a 2-step serial compound dilution in DMSO. Aim for a cell-tolerable DMSO concentration in medium (e.g., 0.1% DMSO).

***Optional:*** Utilize either automated liquid handling systems (e.g., ECHO liquid handler) or prepare plates manually.

## Key resources table

**Table:** 

REAGENT or RESOURCE	SOURCE	IDENTIFIER
**Chemicals, peptides, and recombinant proteins**		

Anti-adherence rinsing solution	STEMCELL Technologies	Cat# 07010
Dimethyl sulfoxide (DMSO)	Sigma-Aldrich	Cat# D5879
DMEM/F-12	Gibco	Cat# 21331046
Dulbecco's Phosphate Buffered Saline (D-PBS)	Sigma-Aldrich	Cat# D8537
Fetal Bovine Serum (FBS) advanced	Capricorn Scientific	Cat# 10-FBS-11 F
Fraunhofer repurposing library	N/A	^26^ and in-house
Gentle Cell Dissociation Reagent	STEMCELL Technologies	Cat# 100-0485
L-glutamine	Capricorn Scientific	Cat# GLN-B
Matrigel	Corning	Cat# 356231
Penicillin-streptomycin (Pen/Strep)	Capricorn Scientific	Cat# PS-B
Selinexor	Selleckchem	Cat# S7252
Trypsin-EDTA	PAN-Biotech	Cat# P10-024100
Y-27632 (Dihydrochloride)	STEMCELL Technologies	Cat# 72304

**Critical commercial kits**		

CellTiter-Glo 3D Cell Viability Assay	Promega	Cat# G9682
mTeSR Plus	STEMCELL Technologies	Cat# 100-0276
STEMdiff Dorsal Forebrain Organoid Differentiation Kit	STEMCELL Technologies	Cat# 08620
STEMdiff Neural Organoid Maintenance Kit	STEMCELL Technologies	Cat# 100-0120

**Experimental models: Cell lines**		

Human iPSC line: ZIPi013-B	ZIP gGmbH,^22^	RRID: CVCL_UF44
Human iPSC line: HHUUKDi009-A	HHU Düsseldorf,^21^	RRID: CVCL_B3T9
Human iPSC line: WISCi004-B	WiCell Research Institute,^23^	RRID: CVCL_C437
Human melanoma cell line: A375	ATCC	Cat# CRL-1619; RRID: CVCL_0132
Human melanoma cell line: Disseminated cancer cells (MelDCCs)	University of Regensburg/Fraunhofer ITEM-R	N/A

**Recombinant DNA**		

LeGO-T2	University Medical Center Hamburg-Eppendorf	Addgene, Cat#27342, RRID: Addgene_27342
pRRL-CMV-GFP-puro	Swiss Institute of Cancer Research	N/A

**Software and algorithm**		

GraphPad Prism 10	GraphPad	http://www.graphpad.com/; RRID: SCR_002798
Kaleido software	Revvity	N/A

**Other**		

10 mL reservoir	Integra	Cat# 4332
6-well plates	Greiner Bio-One	Cat# 657-160
96-well cell culture microplate, CELLSTAR, white	Greiner Bio-One	Cat# 655083
96-well masterblock	Greiner	Cat# 780271
96-well ultra-low attachment plates	Corning	Cat# 7007
AggreWell 800	STEMCELL Technologies	Cat# 34815
Cell culture warming plate	Labotect	N/A
Cell Lifter	Corning	Cat# 3008
Echo 650 liquid handler	Beckman Coulter	N/A
Echo qualified 384-well plates	Labcyte	Cat# 001-16128
EnSight multimode plate reader	Revvity	N/A
Multi-channel micropipettes	Brand	N/A
Reversible Strainer	STEMCELL Technologies	Cat# 27215
Sealing film	Carl Roth GmbH + Co. KG	Cat# KKE1.1
Shaker	Edmund Bühler GmbH	N/A
T75 Flasks	Sarstedt	Cat# 83.39.11.002

## Materials and equipment

**Table:** A375 Complete Medium

Reagent	Final concentration	Volume
DMEM/F12	N/A	88 mL
Fetal Bovine Serum (100×)	10×	10 mL
Glutamine (200 mM)	2 mM	1 mL
Pen/Strep (100×)	1×	1 mL
**Total**	**N/A**	**100 mL**

***Note:*** Store at 2°C–8°C for up to 3 weeks.

**Table:** Organoid Expansion Medium (STEMdiff Dorsal Forebrain Organoid Differentiation Kit)

Reagent	Final concentration	Volume
Basal Medium 2	N/A	243.5 mL
Supplement A (100×)	2×	5 mL
Supplement B (100×)	0.1×	0.25 mL
Pen/Strep (100×)	0.5×	1.25 mL
**Total**	**N/A**	**250 mL**

***Note:*** Store at 2°C–8°C for up to 3 weeks.

**Table:** Organoid Differentiation Medium (STEMdiff Dorsal Forebrain Organoid Differentiation Kit)

Reagent	Final concentration	Volume
Basal Medium 2	N/A	243.5 mL
Supplement A (100×)	2×	5 mL
Supplement C (100×)	0.1×	0.25 mL
Pen/Strep (100×)	0.5×	1.25 mL
**Total**	**N/A**	**250 mL**

***Note:*** Store at 2°C–8°C for up to 3 weeks.

**Table:** Organoid Maintenance Medium (STEMdiff Dorsal Forebrain Organoid Differentiation)

Reagent	Final concentration	Volume
Basal Medium 2	N/A	97.5 mL
Supplement A (100×)	2×	2 mL
Pen/Strep (100×)	0.5×	0.5 mL
**Total**	**N/A**	**100 mL**

***Note:*** Store at 2°C–8°C for up to 3 weeks. Medium is viscous at pipetting. For additional Organoid Maintenance Medium utilize STEMdiff Neural Organoid Maintenance Kit.

## Step-by-step method details

### Cortical organoid generation

**Timing: ≥ 43 days**

This section provides a detailed overview of single handling steps and modifications from the original protocol based on^27^ and STEMdiff Dorsal Forebrain Organoid Differentiation Kit. In this protocol we describe the processing of one individual hCO batch based on 6 × 10^6^ hiPSCs, using one STEMdiff Dorsal Forebrain Organoid Kit which provides sufficient reagents for two wells of an AggreWell 800 microwell culture plate, giving rise to a maximum of 240 hCOs distributed in four 96-well plates.

**CRITICAL:** When working with multiple batches at the same time, keep the cultures always separated and utilize fresh plastic ware to prevent cross-contamination.

***Note:*** We aim to reduce the amount of weekend work and thus schedule organoid formation on Thursdays. All cell culture work needs to be conducted under aseptic conditions within a laminar flow hood.

1. Organoid formation.
  a. On day 0 (Thursday), warm STEMdiff Neural Organoid Basal Medium 1 (referred to as Organoid Formation Medium) up to RT (20°C–25°C) and prepare 4 mL of Seeding Medium by supplementation of 10 μM Y-27632.
  b. Treat two wells of an AggreWell plate with anti-adherence rinsing solution.
  c. Dissociate the prepared hiPSC cultures by incubating with Gentle Cell Dissociation Reagent for 8–10 min at 37°C, to achieve a single cell suspension.
  d. Centrifuge cell suspension at 300 × *g* for 5 min and remove supernatant.
  e. Resuspend the pellet in approximately 3–6 mL of mTeSR Plus medium to achieve a concentration of 1–2 × 10^6^ cells/mL and count cells.
    **CRITICAL:** Accurate and robust counting technique is mandatory to achieve homogenous size of individual hCO batches.
    ***Optional:*** We recommend counting representative 10 μL of cell suspension twice (manual and automated) and calculating the average.
  f. Centrifuge the cell suspension corresponding to 6 × 10^6^ hiPSCs at 300 × *g* for 5 min and remove supernatant.
  g. Resuspend 6 × 10^6^ hiPSCs in 4 mL Seeding Medium and pipet 2 mL per AggreWell to achieve a suspension of 1 × 10^4^ cells/microwell.
    ***Note:*** Ensure even distribution in the well by gentle pipetting.
  h. Centrifuge the plate at 100 × *g* for 3 min.
    ***Note:*** If required, adapt centrifugation settings for acceleration and deceleration to a lower speed, to maintain the cells in the microwells.
    ***Optional:*** Seal plates with parafilm to stabilize the plate lid during the centrifugation. Remove the parafilm afterwards.
  i. Place the AggreWell plate in the incubator at 37°C for 24 h.
    ***Note:*** Observe the morphology under the microscope. Spherical structures should form within the initial 24 h (exemplary image in Figure 1B). For more information see problem 2.

**Figure 1:**
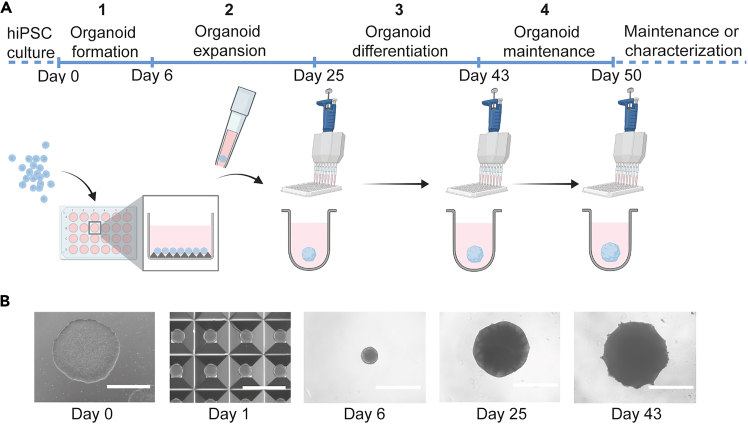
Overview of human cortical organoid (hCO) generation in 96-well plates (A) Schematic overview illustrates stages 1–4 of the hCO protocol, starting from human induced pluripotent stem cell (hiPSC) colonies, via assembly in microwell plates, and manual transfer to 96-well plates. The medium exchange during organoid expansion, differentiation and maintenance is standardized using multichannel pipettes. (B) Microscopic brightfield images illustrate the stages from hiPSCs to hCOs. The scale bars represent 1000 μm. Figure reprinted and adapted with permission from.^1^
  j. On day 1–5, carefully perform daily exchange of 1.5 mL/well Organoid Formation Medium stepwise using 1000 μL pipette.
    ***Optional:*** Carefully remove 750 μL medium in two steps, then add 750 μL of fresh Organoid Formation Medium twice using a 1000 μL pipette.
    **CRITICAL:** The plate must be handled carefully during medium exchanges to prevent uncontrolled movement of hCOs, resulting in spontaneous fusion or tissue damage. For more information see problem 3.
2. Organoid expansion.
  **CRITICAL:** From this stage it is recommended to use wide-bore tips (manually modified through trimming and autoclaving) for the transfer of organoids to maintain structural integrity. If the processing time exceeds 20 min, work on a cell culture warming plate set to 37°C.
  a. On day 6, aggregates should present a spherical morphology with a compact center and clear borders (exemplary image in Figure 1B).
  b. Prepare Organoid Expansion Medium and warm up to RT (20°C–25°C).
    ***Optional:*** Supplement medium with 0.5% Pen/Strep.
  c. Treat one well of a 6-well plate as well as one 50 mL conical tube with anti-adherence rinsing solution labeled “organoids”.
    ***Note:*** Short treatment with anti-adherence rinsing solution is sufficient to reduce the risk of organoid attachment to the surface of vessels or pipette tips.
  d. Unpack a sterile reversible strainer and place it on top of a second 50 mL conical tube, labeled “waste”.
    ***Optional:*** Wet the strainer with 1 mL of DMEM/F-12 before usage.
  e. Label up to four 96-well ultra-low attachment plates with the respective sample IDs.
  f. Collect all aggregates from both AggreWells through the strainer using a 1000 μL pipette with wide-bore tips.
  g. Invert the strainer over the low-attachment tube labeled “organoids” and expel 2–3 mL Organoid Expansion Medium.
  h. Gently swirl the aggregates to create a uniform suspension.
    ***Note:*** Gentle periodic agitation can minimize the risk of spontaneous organoid fusion.
    ***Optional:*** Wash the wells to increase the organoid yield.
  i. Use a 200 μL pipette with wide-bore tips to transfer 10–30 aggregates from the AggreWell into the prepared low-attachment 6-well plate, filled with Organoid Expansion Medium.
  j. Transfer single aggregates from the 6-well in 50 μL medium to each well of a 96-well ultra-low attachment plate.
    ***Note:*** Repeat this step until a maximum of four plates are filled. Follow your treatment layout.
    **CRITICAL:** Leave out any border wells of the plate, which results in maximal 60 transferred aggregates per plate. Fill the border wells with 200 μL D-PBS to avoid evaporation of medium over the culture duration.
    ***Optional:*** Supplement D-PBS with 0.5% Pen/Strep.
  k. Confirm successful single transfer of organoids per well using the EnSight multimode plate reader or similar brightfield imaging device. Place plates covered with a plate lit or in a sterile environment into the imaging device.
    **CRITICAL:** The imaging should be conducted at 37°C or not exceed 20 min at RT (20°C–25°C).
    ***Note:*** If required, repeat the transfer process. For more information see problem 4.
  l. Add additional 80 μL of Forebrain Organoid Expansion Medium to each well using sterile reservoirs and multichannel pipettes with low speed, now achieving a total volume of 130 μL.
    ***Note:*** From this point, conduct liquid handling using a standardized procedure with multichannel pipettes to ensure consistency of dispensing position and speed across samples of the same batch. It is recommended to aspirate and dispense the medium at the well borders to protect the structural integrity of organoids. When multiple batches are processed in parallel, avoid cross-contamination by using fresh reagents for every batch. Account for dead volume in reservoirs by preparing at least 10% excess volume.
  m. Gently place the plates on a level surface of the incubator for two days.
  n. Confirm organoid morphology and intactness as well as absence of contamination using brightfield microscopy before medium exchanges three times a week.
    i. After two days, carefully add 70 μL of fresh medium to each well using the multichannel pipette to achieve a total culture volume of 200 μL.
    ii. For the following days, gently aspirate 100 μL (on Mondays and Wednesdays) and 150 μL (on Fridays) of medium from each well using the multichannel pipette.
    iii. Carefully add 100 μL (on Mondays and Wednesdays) and 150 μL (on Fridays) of fresh medium to each well using the multichannel pipette.
      ***Note:*** Observe morphology and growth over time of cultivation. For more information see problem 5 and problem 6.
3. Organoid differentiation.
  a. On day 25, prepare Organoid Differentiation Medium and warm at RT (20°C–25°C).
    ***Optional:*** Supplement medium with 0.5% Pen/Strep.
  b. Confirm organoid morphology and intactness, as well as absence of contamination using brightfield microscopy before medium exchanges three times a week.
    ***Note:*** In high-contrast brightfield images, organoids should demonstrate ring-shaped neurogenic zones (exemplary image in Figure 1B).
    i. Gently aspirate 100 μL (on Mondays and Wednesdays) and 150 μL (on Fridays) of medium from each well using the multichannel pipette.
    ii. Carefully add 100 μL (on Mondays and Wednesdays) and 150 μL (on Fridays) of fresh medium to each well using the multichannel pipette.
      ***Note:*** Account for dead volume in reservoirs by preparing at least 10% excess volume.
      ***Optional:*** Perform filling of D-PBS in border wells in case of volume loss.
4. Organoid maintenance.
  a. On day 43, prepare Organoid Maintenance Medium and warm at RT (20°C–25°C).
    ***Optional:*** Supplement medium with 0.5% Pen/Strep.
  b. Confirm organoid morphology and intactness, as well as absence of contamination using brightfield microscopy before medium exchanges three times a week.
    i. Gently aspirate 100 μL (on Mondays and Wednesdays) and 150 μL (on Fridays) of medium from each well using the multichannel pipette.
    ii. Carefully add 100 μL (on Mondays and Wednesdays) and 150 μL (on Fridays) of fresh medium to each well using the multichannel pipette.
      ***Note:*** Account for dead volume in reservoirs by preparing at least 10% excess volume.
      ***Optional:*** Perform filling of D-PBS in border wells in case of volume loss.

### Co-cultivation of cortical organoids and melanoma cells

**Timing: Seven days**

In this step, we describe the co-cultivation of hCOs at cultivation day 43 in Organoid Maintenance Medium with prepared 2D maintenance cultures of A375 melanoma cells. All cell culture work needs to be conducted under aseptic conditions within a laminar flow hood.

**Figure 2:**
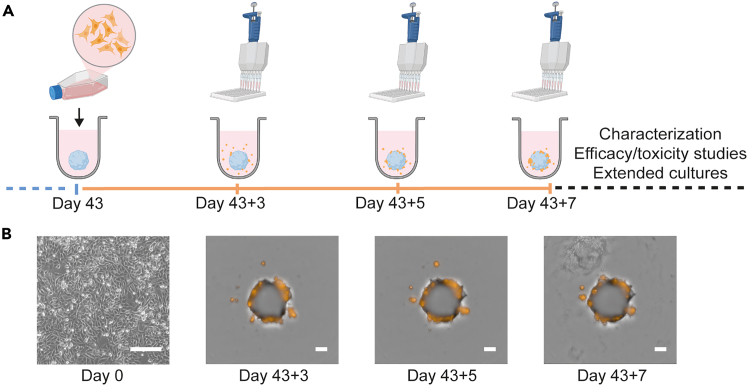
Overview of co-culture procedure to generate melanoma brain metastases (MBM) models (A) Schematic overview illustrating the formation of MBM models in 96-well plates. During the organoid maintenance stage, fluorescently labeled melanoma cells are added to day 43-old hCOs for seven days. Medium exchange is standardized using multichannel pipettes. Here, we demonstrate the colonization of hCOs by RFP-expressing A375 cells, referred to as A375-MBM models. (B) Representative brightfield and fluorescence images of A375 maintenance culture prior to harvesting (day 0), and of A375-MBM models during co-cultivation. The scale bar on day 0 represents 200 μm. Scale bars for remaining images represent 400 μm.

5. On day 43 of organoid culture, detach A375 cells from 2D maintenance cultures using Trypsin-EDTA when ready for passaging. Figure 2B provides an example of A375 cell quality and confluency.
  a. Collect the cell pellet after centrifugation in DMEM/F12 and thoroughly resuspend the single-cell suspension for counting.

**CRITICAL:** Accurate counting technique is mandatory to achieve homogenous MBM batches.

***Optional:*** We recommend counting twice (manual and automated) and calculating the average.

6. Prepare a single-cell suspension of melanoma cells in fresh Organoid Maintenance Medium resulting in 1 × 10^4^ cells per 150 μL added to each hCO.
  ***Note:*** Account for dead volume in reservoirs by preparing at least 10% excess volume.
  a. Gently aspirate 150 μL of medium from each well of 96-well plates containing hCOs using the multichannel pipette.
  b. Mix the melanoma cell suspension thoroughly and transfer it into the reservoir.
  c. Carefully add 150 μL of the prepared melanoma cell suspension to each well using the multichannel pipette.
    **CRITICAL:** Gentle periodic agitation in reservoirs supports a homogenous distribution of melanoma cells across plates.
  d. Centrifuge the plates at 100 × *g* for 3 min.
    ***Optional:*** Seal plates with parafilm to stabilize the plate lid during centrifugation. Afterwards, remove the parafilm.
7. Place the plates back into the incubator and let co-cultures (referred to as MBM model) settle for two days.

***Note:*** The addition of cancer cells is considered day 43+0. If you follow the proposed timeline, day 43 is a Friday and you can continue with the usual organoid maintenance cycle of medium changes for the next seven days.

8. Perform regular medium changes using the multichannel pipette.
  a. On day 43+3, gently aspirate 100 μL of medium from each well.
  b. Carefully add 100 μL of fresh medium to each well.
  c. On day 43+5, gently aspirate 100 μL of medium from each well.
  d. Carefully add 100 μL of fresh medium to each well.
  e. On day 43+7 (corresponding to day 50), use MBM models for downstream analysis.

**CRITICAL:** Observe morphology and fluorescence using the EnSight multimode plate reader or similar imaging device (exemplary images in Figure 2B). For more information check problem 6 and problem 7.

***Optional:*** Continue the procedure including regular medium exchange for extended long-term cultures. Perform filling of D-PBS in border wells in case of volume loss.

### Morphometric analysis based on bright-field and fluorescence live imaging

**Timing: ∼10–20 min per plate**

This step provides information about regular monitoring of morphological parameters (e.g., organoid size and roundness) as well as the fluorescence intensity of MBM models with or without compound exposure.

***Note:*** We describe the imaging procedure using the EnSight multimode plate reader and Kaleido software. Similar live- and fast- brightfield and fluorescence imaging devices followed by multi-parametric analysis pipelines may be suited.

9. Place plates covered with a plate lit or in a sterile environment into the imaging device.

**CRITICAL:** The imaging should be conducted at 37°C or not exceed 20 min at RT (20°C–25°C).

10. Image all samples in brightfield and the corresponding channel of your fluorescent protein of choice with multiple planes.

***Note:*** We used seven planes spanning approximately 1800 μm in brightfield and fluorescence channels as followed: Brightfield (excitation wavelength: 735 nm; excitation power: 4%; exposure time: 4 ms), RFP (excitation wavelength: 525 nm; excitation power: 100%; exposure time: 30 ms) GFP (excitation wavelength: 465 nm; excitation power: 4%; exposure time: 4 ms).

***Optional:*** We further performed z-stack processing for representative images providing spatial information beyond single-plane imaging.

11. Perform batch processing of representative single image planes.
  a. Segment the organoid as the largest round object in the brightfield channel.
  b. Analyze the morphology of hCO.
  c. Analyze the corresponding fluorescence intensity within the organoid region.

**Figure 3:**
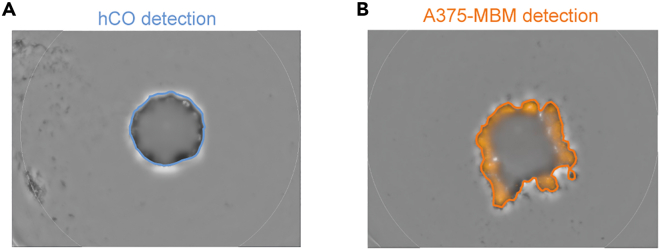
Exemplary morphometric analysis of hCOs and MBM models based on brightfield and fluorescence imaging (A) The blue line indicates the segmentation of the considered hCO region for downstream morphological analysis. (B) The orange line indicates the considered A375-MBM model region for analysis of fluorescence intensity within the organoid region, representing RFP-expressing colonized melanoma cells. Figure reprinted and adapted with permission from.^1^

**CRITICAL:** The analysis should exclusively account for fluorescently labeled cancer cells within the detected organoid region but not free-floating cancer cells.

***Note:*** We recommend the spheroid detection of Kaleido software for batch processing with the object detection settings (tuning of brightness limit: 0.6, flatfield correction scale of brightfield: 0, minimum object size: 100,000 μm^2^, minimum contrast requirements: very relaxed, ring-shaped objects: yes, largest objective, only: yes) and well detection settings (channel: automatic, exclude well margin 400 px, mode: small well, well dimensions: automatic, well shape: round, well diameter: 6.3 mm). For exemplary images see Figure 3. Modification may be required for your experimental setup.

### Pre-screening quality control

**Timing: ∼10–20 min per plate + 2–3 additional days (for new co-culture models only)**

This section describes quality control measures for disease phenotypes and model homogeneity, which should be in place prior to continuing with drug screening approaches.

***Note:*** The performance of hiPSC lines to differentiate into neuroectoderm, in particular to develop into hCOs, needs to be assessed by routine gene and protein expression analysis, as shown in.^1^

12. When establishing new brain metastasis models, investigate the metastatic signature to confirm the model’s disease phenotypes.^1^^,^^18^^,^^20^
  a. Analyze proliferation and growth pattern of metastases on the hCO tissue.
    i. Confirm the increase of metastatic outgrowth during co-cultivation over time by consecutive brightfield and fluorescence imaging using the EnSight multimode plate reader or a similar imaging device.
    ii. Confirm the presence of invasive cancer cells as well as colony formation in fixed MBM models on day 50 using histology, immunohistochemistry or whole-mount staining protocols followed by imaging, e.g., confocal or light-sheet microscopy.
      ***Note:*** Expect melanoma cells to develop into nodule-like metastatic structures, accompanied by the invasion of individual cells and small clusters of cells into the hCO tissue.
  b. We also recommend analyzing differential gene and protein expression related to brain metastasis-associated biological processes in MBM models compared to hCOs.
    ***Note:*** Depending on cancer cell lines, such biological processes may involve epithelial-to-mesenchymal transition factors (e.g., *TWIST1*), extracellular matrix reorganization (e.g., *FN1*) or alterations in immune modulators (e.g., *CXCL10*). Early colonization events in A375-MBM models are marked by the presence of invasive-/mesenchymal-like melanoma cells and the emergence of astrocyte-like cell populations at the contact sites.
    i. Collect dry pellets on day 50, store at −80°C and process for gene expression analysis, such as mRNA sequencing or RT-qPCR.
    ii. Fix samples on day 50 for histology, immunohistochemistry or whole mount staining protocols followed by imaging, e.g., confocal or light-sheet microscopy.
    iii. Collect sample supernatant on day 50, store at −20°C and process further for secretome analysis, e.g., ELISA.
13. Before drug testing, assess hCO morphology and MBM growth kinetics to ensure homogeneity among samples within each batch and assay sensitivity.
  a. Perform live- and fast-brightfield and fluorescence imaging using the EnSight multimode plate reader or any similar imaging device combined with spheroid detection analysis to compare the size as one parameter of morphology and the fluorescence intensity as a proxy of the amount of metastatic outgrowth.
  b. As an indicator for precision and reproducibility across individual batches, calculate the coefficient of variance (CV) of both measures and aim for a CV ≤ 10%.
  c. When you establish new co-culture systems, observe whether the overall fluorescence intensity of co-cultures at day seven is suitable for drug screening.

**CRITICAL:** Calculating the signal-to-noise ratio, as the difference between the fluorescence intensity of MBM models and the autofluorescence of hCOs, provides information about signal intensity and assay sensitivity. If the difference is too small, resulting in a narrow assay window, the assay cannot reliably distinguish between hits with anti-metastatic activity and random fluctuation during drug testing. For more information see problem 7.

### Compound screening

**Timing: 3–6 days (including incubation)**

In this section, the preparation of final compound concentrations and treatment procedure for hCOs and MBM models is described. All cell culture work needs to be conducted under aseptic conditions within a laminar flow hood. If the processing time exceeds 20 min, work on a cell culture warming plate set to 37°C. Consider time-course staggering when managing large-scale drug screens and multiple batches at the same time.

**CRITICAL:** Compounds may be temperature- and light-sensitive.

14. Prepare final concentrations of compounds freshly in Organoid Maintenance Medium.
  ***Note:*** When treating multiple wells equally for single-concentration screening, we recommend preparing final compound concentrations in falcon tubes before transferring to reservoirs. To prepare serial dilutions in medium, we recommend using 96-well masterblocks.
  a. Adjust all conditions to equal final concentrations of DMSO (e.g., 0.1% DMSO) and mix compounds well to achieve uniform solutions.
    ***Note:*** Follow the layout and always account for dead volume in reservoirs and masterblocks by preparing at least 10% excess volume.
15. Perform compound treatment using pre-screened batches at cell culture day 50.
  a. Gently aspirate all media from each well using multichannel pipette tips by tilting the plate in a 30–45 degree angle.
  b. Carefully dispense 100 μL freshly prepared compound dilutions from reservoirs or masterblocks to each well using multichannel pipettes.
    **CRITICAL:** Observe morphology under the microscope. For more information see problem 6.
  c. Place plates in the incubator for at least 24 h.
    ***Note:*** For compound screening of larger compound sets we apply compounds once and incubate for three days (72 h). For compound validation studies (e.g., concentration-response curves), compounds can be refreshed every day. We have performed compound replenishment for up to six consecutive days (144 h). Compound incubation exceeding 72 h without medium replenishment may lead to nutrient depletion.
    **CRITICAL:** Avoid cross-contamination of compounds, in particular during compound replenishment experiments.

### Cellular viability readout

**Timing: 10–60 min per plate**

**Figure 4:**
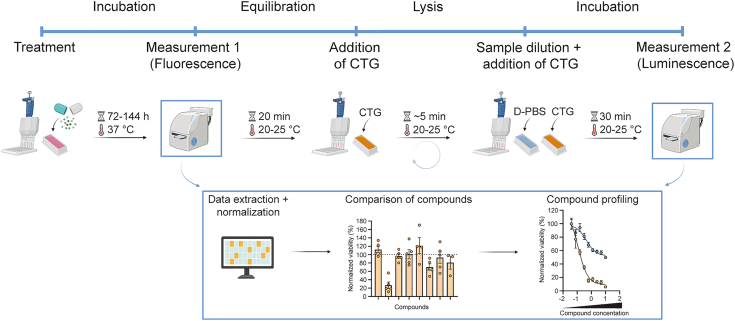
Overview of compound treatment, screening assay and analysis Incubation of compounds takes place for 72–144 h depending on individual research questions. After measurement 1 (fluorescence), perform CellTiter-Glo (CTG) assay for measurement 2 (luminescence). Extract data from spreadsheets for further data analysis and visualization.

This step details the cellular viability assay of MBM models based on fluorescence intensity or hCOs based on ATP-dependent luminescence to address anti-metastatic activity and neurotoxic effects of compounds (Figure 4). Consider time-course staggering when managing large-scale drug screens and multiple batches.

16. Measure fluorescence intensity as an indicator of MBM cellular viability.
  a. Image full plates of MBM models using the EnSight multimode plate reader or similar imaging devices.
    ***Note:*** Also image a representative plate of hCOs in phenol red-containing Organoid Maintenance Medium to determine the autofluorescence intensity.
  b. Analyze the fluorescence intensity using the Kaleido software or equivalent batch processing pipeline.
  c. Export raw intensity data for all wells into a spreadsheet.
17. Perform CellTiter-Glo 3D Cell Viability Assay in treated hCO cultures.
  **CRITICAL:** The assay is temperature- and light-sensitive.
  a. Thaw reagents overnight (16–18 h) at 4°C and warm to RT (20°C–25°C).
  b. Adjust cell culture plates to RT (20°C–25°C) for 20 min.
  c. Add 100 μL of CellTiter-Glo reagent directly to 100 μL organoid suspension using reservoirs and multichannel pipettes.
  d. Induce lysis through repetitive pipetting equally in all wells. Then place plates on a shaker at 60 rpm for 5 min.
    **CRITICAL:** Observe samples under the brightfield microscope and repeat the pipetting and shaking step until tissues are fully lysed. Homogenous tissue lysis of samples is mandatory for the intracellular ATP-dependent readout.
  e. We recommend diluting the lysates 1:10 in D-PBS by transferring 10 μL of lysates into 90 μL of D-PBS prepared in a 96-well white-bottom plate.
    **CRITICAL:** Consider the detection limit of your experimental setup and adapt to respective dilution ratios.
  f. Add again equal amount of CellTiter-Glo reagent directly to the organoid suspension using reservoirs and multichannel pipettes.
    ***Note:*** If you follow our recommendation, you add an equal amount of 100 μL.
  g. Seal the plates protected from light and incubate on a shaker for 5 min at 60 rpm.
  h. The luminescence signal is established after further incubation for 25 min at RT (20°C–25°C).
  i. Record luminescence using the EnSight multimode plate reader or equivalent device.
  j. Export raw intensity data for all wells into a spreadsheet.

### Analysis and data interpretation

**Timing: ∼30 min per plate**

18. Normalize fluorescence and luminescence data using controls.
  a. Calculate the average autofluorescence intensity of hCOs in medium and subtract the value from all fluorescence values of MBM samples.
  b. Set the average fluorescence or luminescence intensity of negative control wells as 100% cellular viability.
  c. Calculate the relative cellular viability of each compound-treated well as a percentage of the control range.
19. Generate concentration-response curves.
  a. Plot the normalized viability data vs. compound concentration using GraphPad Prism or similar software.
  b. Fit a nonlinear regression model (e.g., sigmoidal concentration-response equation, variable slope, four parameters).
  c. Extract pharmacology parameters (e.g., half inhibitory concentration (IC_50_), maximal efficacy (E_max_)) to compare compound candidates and in relation to positive controls.

## Expected outcomes

The protocol involves multicellular 3D screening models that enable targeting of colonized cancer cells in the metastatic niche tissue. In addition, the miniaturized assay readouts do not require complex handling procedures and reduce both, amount and time of data acquisition and processing.

**Figure 5:**
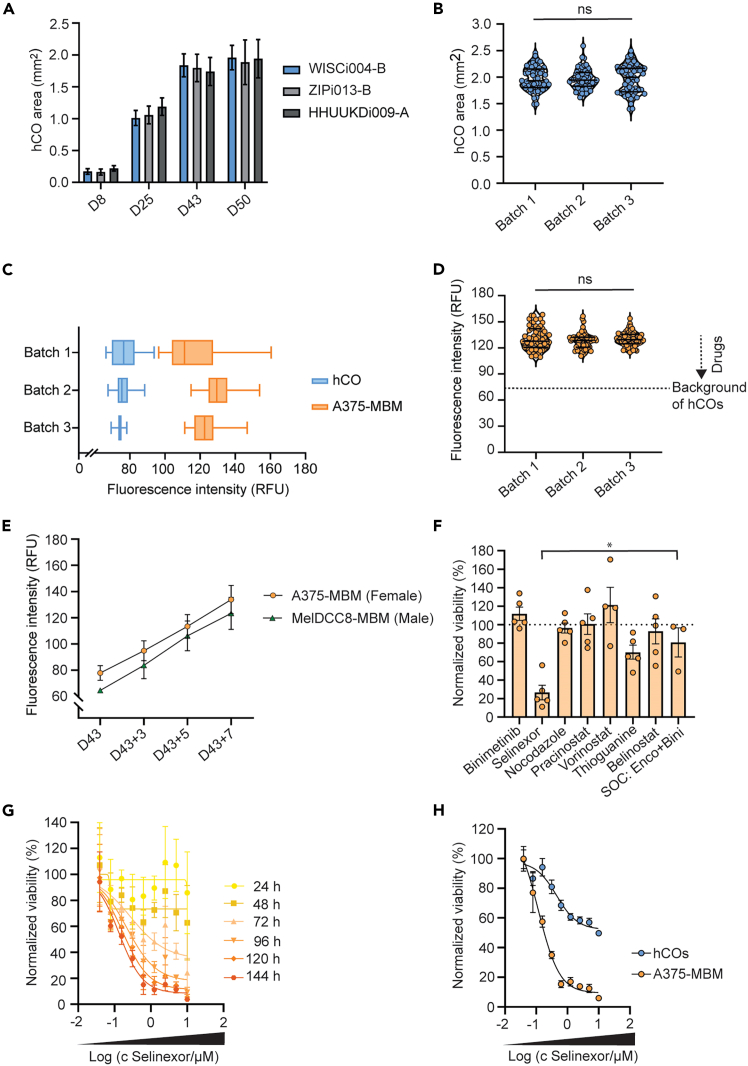
Robust generation of hCOs and MBM platforms for the validation of drug efficacy and safety (A) Robust differentiation and increase of hCO area using hiPSC lines WISCi004-B, ZIPi013-B and HHUUKDi009-A (n ≥ 57 hCOs from each batch per time point). Data are presented as mean ± SEM. (B) Consistent mean hCO area at day 50 (n ≥ 58 hCOs per batch). Data are presented as median and lower/upper quartile. Statistical analysis was performed using one-way ANOVA, followed by Tukey’s multiple comparisons test. ns, not significant. (C) Box and whiskers (min to max) plot of the fluorescence intensity presenting the assay signal of A375-MBM models after seven days compared to the autofluorescence of hCOs (considered the noise) (n ≥ 58 samples per batch). (D) Consistent fluorescence signal and stable assay window to test drugs reducing viability of colonized A375 cells. Reproducibility across three independent A375-MBM batches after seven days (n ≥ 58 samples per batch) with a mean CV of 7.92% ± 1.95%. Data are presented as median and lower/upper quartile. Statistical analysis was performed using one-way ANOVA, followed by Tukey’s multiple comparisons test. ns, not significant. (E) Comparative time course analysis of co-cultures, using either A375 melanoma cell line (female) matched with hCOs derived from hiPSC lines WISCi004-B (female) (n = 6 independent MBM batches), or patient-derived MelDCC8 cell line (male) matched with hCOs derived from HHUUKDi009-A (male) (n = 30 MBM samples), demonstrates the robustness of the workflow. Data are presented as mean ± SD. (F) Fluorescence-based cellular viability of A375-MBM models treated with compounds at 10 μM for 72 h (n = 3–5 experiments). Data are presented as mean ± SEM. Statistical analysis was performed using one-way ANOVA, followed by Tukey’s multiple comparisons test. ∗*p* = 0.0179. (G) Concentration- and time-dependent effect of hit compound selinexor on fluorescence-based cellular viability of A375-MBM models (n = 3 technical replicates per condition). Data are presented as mean ± SD. IC_50_: 0.13 μM. (H) Concentration response of selinexor in A375-MBM models and hCOs. Viability is assessed based on the fluorescence intensity or ATP-based 3D CellTiter-Glo assay after 6 days of treatment (n = 2 experiments per condition). Data are presented as mean ± SEM. Figure reprinted and adapted with permission from.^1^

Starting with high-quality hiPSC cultures, the described workflow generates viable 3D cortical tissues of increasing size. The robustness of our procedure was validated across three healthy hiPSC cell lines (Figure 5A) and enables the generation of homogenous and screening-compatible batches of up to 240 hCOs (Figure 5B).

During the seven days of co-culture, A375 melanoma cells attach to, colonize and proliferate in the hCO tissues. Metastatic outgrowth is indicated by increasing fluorescence intensity over time. The signal-to-noise ratio reveals whether this signal can be distinguished from the autofluorescence with a high degree of confidence (Figure 5C). The standardized assay conditions enable high reproducibility and a stable assay window, with a mean CV of 7.92% ± 1.95%. (Figure 5D). Importantly, the protocol is compatible with patient-derived samples, such as disseminated cancer cells isolated from lymph node biopsies of melanoma patients (Figure 5E). In addition, the protocol allows for the generation of male and female MBM models. Downstream analysis of the MBM models confirmed the colonization, a nodular growth pattern and a metastasis signature at gene and protein level.^1^

The MBM assay is suitable to validate and prioritize compounds targeting metastatic melanoma cells in a brain niche context.^1^ We validated the anti-metastatic activity of drug candidates, identified in an anti-cancer repurposing drug screen using 2D cell cultures of melanoma and disseminated cancer cells. One drug in particular, selinexor, showed potent anti-metastatic activity in MBM models, and outperformed the current standard of care (Figure 5F). In addition, the MBM assay allows for general hit profiling by assessing the concentration-response profile (Figure 5G), as well as testing for drug selectivity and neurotoxic off-target effects (Figure 5H).

## Quantification and statistical analysis

For pre-screening quality control, the signal-to-noise ratio is calculated by subtracting the mean hCO autofluorescence signal from the mean MBM signal and dividing this value by the standard deviation of the hCO autofluorescence. To investigate the intra- and inter-batch variability, calculate the CV in GraphPad Prism by dividing the sample standard deviation by the mean of replicates and multiplying by 100%.

For drug screening, fluorescence and luminescence intensity values were normalized to relative negative controls, in this case 0.1% DMSO, defined as 100% viability.

Relative viability was calculated based on the following equation:

$$Normalized\;viability\;\left(\%\right)=\frac{Intensity\;\left(sample\right)}{Intensity\;\left(mean\;of\;negativ\;control\right)}\times 100\%$$

We plotted normalized viability data against log-transformed concentrations using [log(inhibitor) vs. response - variable slope] option in GraphPad Prism. We extracted activity values such as the IC_50_ and E_max_ along with the goodness-of-fit and confidence interval.

## Limitations

While this procedure enables anti-metastatic activity testing of compounds in a human multicellular MBM model, several limitations should be considered. The hCO tissue contains neuroectoderm-derived cells, thus representing a highly relevant tumor microenvironment to study brain metastasis. Nevertheless, important immune, vascular and stromal compartments are lacking, which limits the platform’s applications to certain disease pathogenesis and compound classes. All data were derived from allogeneic co-cultures using one commercially available melanoma cell line and three patient-derived melanoma cell lines, together with three healthy hiPSC lines and may not fully represent the generalizability of the approach. We expect similar outcomes for other primary cancer entities with brain tropism, such as (disseminated) lung and breast cancer cells, and the generation of autologous models, but have not yet tested the outcome with this protocol. The scalability of the platform was demonstrated, generating 240 samples per batch. We utilized the platform as validation assay for a subset of hit compounds, but not for a high-throughput drug screening of hundreds of compounds. The proposed protocol is not yet integrated in a fully automated liquid handling system, which likely influences speed, cost and the reproducibility of model generation and assay screening. While investigating the CV and signal-to-noise ratio, calculating important parameters such as the Z′ factor could further increase the statistical robustness of the assay platform. To gain mechanistic insights into drugs, we recommend additional endpoint assays, since both measurements suggested here account for cytotoxic effects in MBM models or hCOs only.

## Troubleshooting

### Problem 1

Inconsistent response to compounds, in particular positive control.

### Potential solution

Prepare fresh stocks using fresh DMSO, aliquot and store according to the manufacturer’s guidelines.

### Problem 2

hiPSCs do not form spherical structures.

### Potential solution

Cultivate your hiPSC cells in accordance with the ISSCR guidelines.^28^ Double-check the morphology, pluripotency and growth pattern of your hiPSC line. For a representative image of high-quality hiPSC cultures see Figure 1B.

### Problem 3

**Figure 6:**
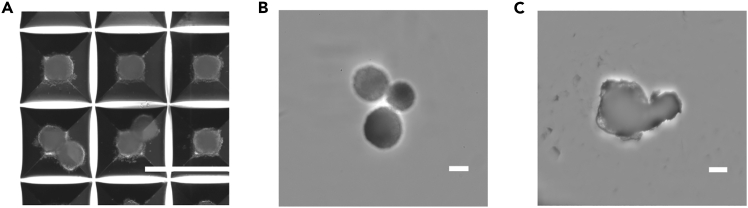
Representative images of inaccurate organoid handling (corresponds to troubleshooting section) (A) Exemplary image of spontaneous organoid fusion caused by incautious and harsh handling of AggreWell plate. The scale bar represents 1000 μm. (B) Exemplary image demonstrates the transfer of multiple aggregates per well of 96-well plates. Scale bar represents 200 μm. (C) Exemplary image of deformed hCO without intact tissue architecture caused by incautious and harsh medium exchange. The scale bar represents 400 μm.

Aggregates spontaneously fuse within AggreWells (see Figure 6A).

### Potential solution

Reduce vibrations in your incubator, during transfer and medium change. Dispense medium slowly to the well borders. This will keep the aggregates in the microwells.

### Problem 4

Aggregates transfer resulted in multiple organoids per well (see Figure 6B).

### Potential solution

If you detect more than one hCO per well, it is recommended to fully remove all aggregates and medium from the well and transfer again.

### Problem 5

hCOs do not grow over time of cultivation.

### Potential solution

Follow the storage and expiration guidelines of the reagent’s manufacturers. Check for potential contamination to provide sufficient nutrients. Double-check the pluripotency and growth pattern of your hiPSC line.

### Problem 6

hCOs or MBM models show deformation or disrupted tissue architecture (see Figure 6C).

### Potential solution

Reduce cellular stress by conducting medium exchange at low speed. Avoid any contact with samples during medium exchange and double-check the position of pipette tips at the well border. Do not let samples dry out and cover them immediately with fresh liquid. If the processing time per plate exceeds 20 min, work on a cell culture warming plate set to 37°C.

### Problem 7

Cancer cells do not colonize hCOs.

### Potential solution

Repeat the centrifugation step using a balanced centrifuge. Double-check the growth rate of your routine cell culture and whether your cancer cells tolerate Organoid Maintenance Medium. In addition, investigate relevant characteristics of your cancer cell line, such as the degree of brain-tropism.

## Resource availability

### Lead contact

Further information and requests for resources and reagents should be directed to and will be fulfilled by the lead contact, Ole Pless (ole.pless@itmp.fraunhofer.de).

### Technical contact

Technical questions on executing this protocol should be directed to and will be answered by the technical contact, Kim Krieg (kim.krieg@itmp.fraunhofer.de).

### Materials availability

This protocol did not generate new unique reagents.

### Data and code availability

This paper did not generate new datasets.

## Acknowledgments

This work was supported by the 10.13039/501100001659German Research Foundation (10.13039/501100001659Deutsche Forschungsgemeinschaft, 10.13039/501100001659DFG), SFB TRR 305, project no. 429280966 (to O.P.), and by a short-term scientific mission grant from COST Action CA20140 ‘‘CorEuStem’’ (to K.K.). We thank the department of Personalized Tumor Therapy of the Fraunhofer Institute for Toxicology and Experimental Medicine ITEM-R and the Department for Experimental Medicine and Therapy Research at the University of Regensburg for providing patient specimens. In addition, we thank Norbert Garbow (Revvity) for technical support with the customized spheroid detection analysis. Some figures were created with BioRender.

## Author contributions

Conceptualization, K.K. and O.P.; data curation, K.K., F.-Z.R., and P.K.; formal analysis, K.K., F.-Z.R., and P.K.; funding acquisition, K.K. and O.P.; investigation, K.K., F.-Z.R., A. Wittich, P. K., and A. Weller; methodology, K.K.; project administration, K.K. and O.P.; resources, O.P.; software, K.K.; supervision, K.K. and O.P.; validation, K.K., F.-Z.R., A. Wittich, P.K., and A. Weller; visualization, K.K., F.-Z.R., A. Wittich, and A. Weller; writing – original draft, K.K.; writing – review and editing, K.K., F.-Z.R., A. Wittich, A. Weller, and O.P. All co-authors contributed to the editing and discussion of the manuscript and approved the final version.

## Declaration of interests

The authors declare no competing interests.
