# Supplementary material for: Protocol for validating drug efficacy and safety in scalable 96-well platforms of human cortical organoids and melanoma brain metastases
Source: STAR Protoc. 2026 May 19;7(2):104568. doi: 10.1016/j.xpro.2026.104568 (PMC13213298; doi:10.1016/j.xpro.2026.104568)
Supplement: Document S1. Figure 1 [file mmc1.pdf]

Supplemental Information

Supplemental figures and legends

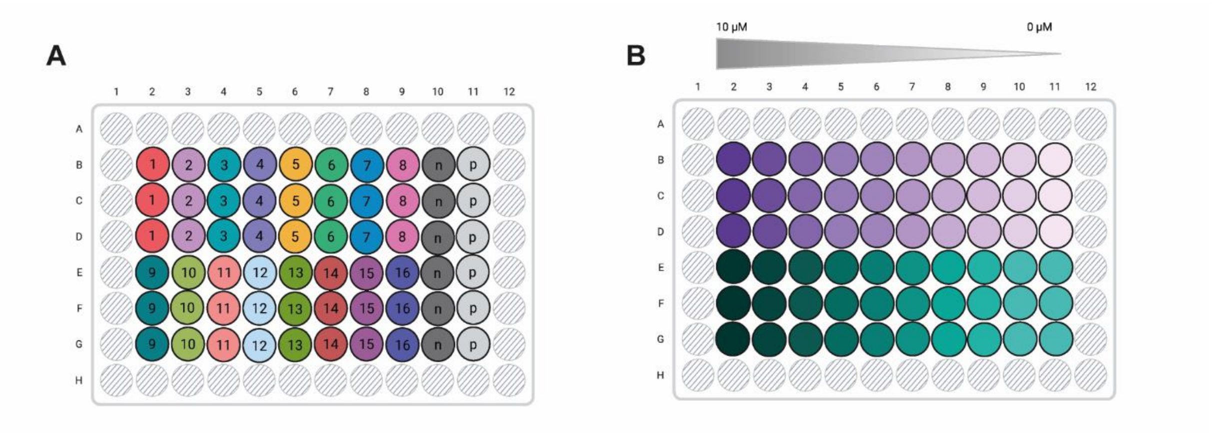

Figure S1: Illustrations of potential plate layouts for compound treatment in 96-well plates, related to Figure 4.

(**A**) Exemplary layout of a compound screen using 16 different compounds in single concentration (here 10 µM), with each three technical replicates. n: negative control, p: positive control. (**B**) Exemplary layout of a concentration-response titration using two compounds, each in three technical replicates. Column 11 (0 µM) refers to the negative control (here 0.1% DMSO).
